# Product News

**DOI:** 10.1155/S1463924683000541

**Published:** 1983


Journal of Automatic Chemistry, Volume 5, Number 4 (October–December 1983), pages 213–220

### Product News

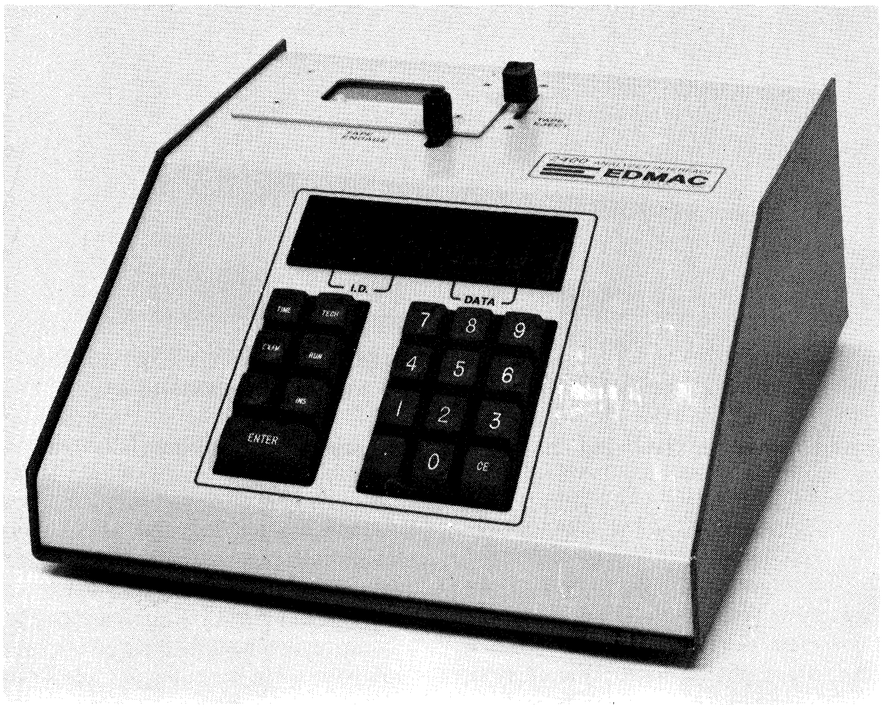

*The Model 2400 Identafacer System: a data-entry terminal for clinical laboratories. The 2400 automatically provides test result tagging for a variety of scales, counters, measuring devices and analysers; it accepts serial, parallel and analogue inputs in ASCII, BCD or Binary data stream formats. Full information from EDMAC Corporation, 7500 Main Street, PO Box 750, Fishers, New York 14453, USA; tel.: 716 924 4000.*

Circle No. 1 on Reader Enquiry Card

### UV/vis spectrophotometer

The new double-beam DMS 100 UV-visible spectrophotometer offers a number of advanced features, together with a flexible repertoire of visual displays via a video monitor. Spectra can be drawn in 'real time' and simultaneously stored in memory; they can then be reformatted on the screen, viewed in a different photometric mode, or overlaid for additional clarity (such as combining the absorbance and second derivative scans). The operator can zoom in on an area of interest, expand the amplitude of the trace, or change the photometric scale by varying both expansion and offset. The results of any of this work can be fed to a video printer and/or a digital printer/plotter.

Varian's DMS 100 also includes a 'Safe Memory' option which stores methods for quick start-ups. This module stores 15 complete set-ups including keyboard status, parameter values and a programmed base-line.

A wide range of further options becomes available if the Varian DS 15 Command Station is substituted for the video monitor: scans can be stored on permanent floppy disc memory and fed back to the spectrophotometer at any time, and manipulated in the same way as a freshly generated trace.

*For full details of the DMS 100 and the DS 15 write to Varian AG, Steinhauserstrasse, CH 6300 Zug, Switzerland.*

Circle No. 2 on Reader Enquiry Card

### Flue gas

A new model of the Anagas Electronic flue-gas analyser has been announced. The new model is equipped with a self-draining facility so it can be used for continuous testing without a build-up of condensation within the unit. A low-cost conversion service is available for existing Anagas users wishing to upgrade their instruments.

*Details from Colwick Instruments Ltd, Anagas House, Station Road, Carlton, Nottingham NG4 3AU, UK. Tel.: 0602 616272.*

Circle No. 3 on Reader Enquiry Card

### Characterizing clay minerals

A new report from Perkin-Elmer details the use of the company's microcomputer-based DTA 1700 differential thermal analysis system for characterizing clay minerals. These characterizations are important to the ceramics, pulp and paper, minerals, fossil fuel, catalysts and polymer industries. The DTA 1700 is compatible with Perkin-Elmer's Thermal Analysis Data Station.

*For further information contact Perkin-Elmer Ltd, Post Office Lane, Beaconsfield, Buckinghamshire HP9 1QA, UK. Tel.: 04946 6161.*

Circle No. 4 on Reader Enquiry Card

### 24 h solvent alarm

A monitor to warn of the potentially dangerous build-up of industrial and commercial solvent vapour is being marketed in the UK by Detectawl (Gastec) of Milton Keynes. The mains-operated 'Gaztell' equipment, manufactured by Autoclimate of Birmingham, will detect vapours from all the chlorinated and fluorinated solvents used as degreasers and dry-cleaning agents (for example perchloroethylene, 111 trichloroethane and trichlorotrifluoroethane).

The wall-mounted Gaztell (which is about the size of a telephone) samples the atmosphere every 2 min. Dual flashing lights and an alarm operate when the vapour concentration exceeds a pre-set threshold. If the master sensing unit is located in a normally unoccupied area it can be linked to a remote alarm, also operated from mains electricity, with identical visual and audible warnings. The power-supply is 240V/110V, 50/60Hz.

*Detectawl (Gastec) is at Unit 61, Garamonde Drive, Wymbush, Milton Keynes MK8 8DE, UK. Tel.: 0908 568076.*

Circle No. 5 on Reader Enquiry Card

### Auto-injector luminescence system

In luminescent assays, rapid and thorough mixing of reagents such as luciferase or luminol with the sample is critical to achieving low variability. Turner Designs' motorized auto-injector system for luminometers has reduced CVs to 1% or less, determined with ATP standards. The injector volume can be adjusted from 50 to 500 µl, and optional syringes permit volumes of 25 µl or less. The typical injection speed is less than 0·5 s; up to three different reagents can be injected into one cuvette. With a count time of 15 s, more than 100 samples can be processed per hour.

*Details from Techmation Ltd, 58 Edgware Way, Edgware, Middlesex HA8 8JP, UK. Tel.: 01 958 3111.*

Circle No. 6 on Reader Enquiry Card

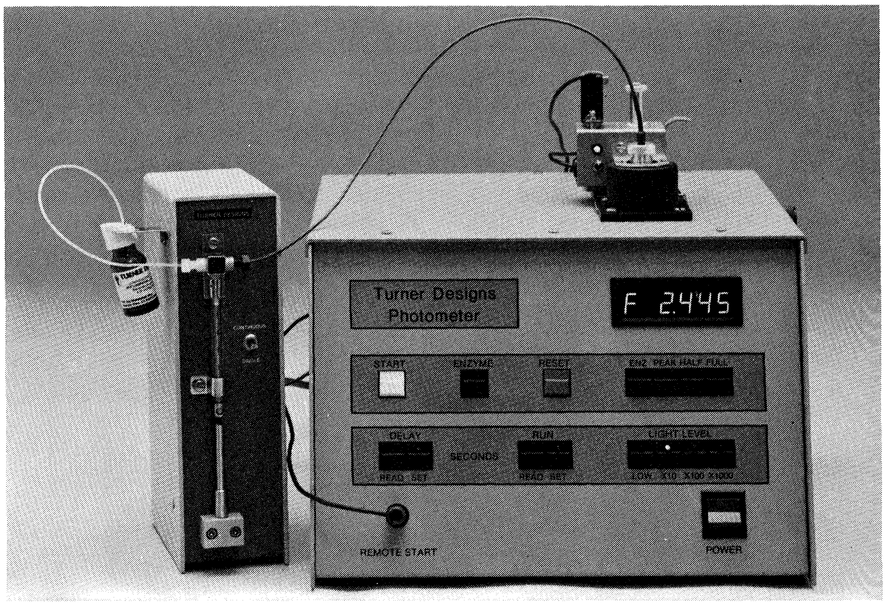

*The Model 20-031 injector system which can be adapted to the existing Turner photometer. The exclusive British Isles distributor is Techmation Ltd.*

### Vacuum heating technology

Following demands for improved performance by users of vacuum ovens, Faircrest Engineering Ltd has developed a method of internal electric heating which overcomes the problem of corona discharge. The new technology, which should be of interest to those using vacuum equipment, is being used by the company for all their deliveries of heated vacuum chambers and replaces the commonly used and complex methods of superheated steam, hot oil or external heating. Temperatures range from ambient to 700°C and vacuum from atmospheric to $10^{-6}$ torr.

*Further details from Faircrest Engineering Ltd, 4 Union Road, Croydon, Surrey CR0 2XX, UK. Tel.: 01 684 1422.*

Circle No. 7 on Reader Enquiry Card

### Luminometry automation

New modular developments have been announced by Techmation in Turner Designs Model 20 photometer/luminometer systems. All of the systems are designed to be compatible with existing components for increased versatility and automation of clinical and quality-control luminescence applications. A news-sheet from Turner Designs describes an auto-injector system for consistent repeat injections, a continuous-flow cuvette, and a three-reagent injector tube assembly. Also shown are data-reduction systems involving a computer interface.

*Copies from Techmation Ltd, 58 Edgware Way, Edgware, Middlesex HA8 8JP, UK. Tel.: 01 958 3111.*

Circle No. 8 on Reader Enquiry Card

### Chlorophyll 'in vivo' and 'in situ'

Accessory kits from Techmation Ltd convert the Turner Field Fluorometer into a portable chlorophyll-measuring instrument, capable of detection down to five parts per billion. The Model 10 fluorometer can be used on land or from a boat. Algae, phytoplankton, diatoms etc. can be measured directly on site without chemical extraction procedures. The water is pumped through the field fluorometer and data can then be read or recorded continuously.

The fluorometer can also be used for oil-in-water and water flow measurements using fluorescent dyes.

*An illustrated brochure is available from Techmation Ltd, 58 Edgware Way, Edgware, Middlesex HA8 8JP. Tel.: 01 958 3111.*

Circle No. 9 on Reader Enquiry Card

### Science Exchange Service

The Walton-based Science Exchange Service buys and sells a range of instruments for research and routine laboratories. The idea is simply that original owners can get rid of unwanted machines—the sale gives them funds for new purchases, and second-hand equipment is available to scientists who cannot afford new instruments. The Service handles over 80 types of machine to full manufacturer's specification and with a warranty—amino-acid analysers, UV monitors, gas chromatographs and so on.

*Details from the Science Exchange Service at Rutherford House, 43 Terrace Road, Walton-on-Thames, Surrey KT12 2SP, UK.*

Circle No. 10 on Reader Enquiry Card

### Instruments for clinical chemistry labs

Beckman has put together a folder on 'stat', or emergency, uses for eight of their instruments. The folder covers machines for use during night calls, as back-ups to a main analyser, and those which will cope with early morning or out-of-run samples. Each instrument is described with an illustrated information sheet; Beckman list the Model 42 clinical chemistry system; glucose, blood urea and creatinine stat analysers, analysers for electrolyte determination; and their Airfuge ultracentrifuge and Microfuge centrifuge.

*Folders are available from Beckman–RIIC Ltd, Progress Road, Sands Industrial Estate, High Wycombe, Buckinghamshire, UK. Tel.: 0494 41181.*

Circle No. 11 on Reader Enquiry Card

### Moisture determination

Baird & Tatlock are now offering a unique instrument for fast and accurate measurement of moisture in solid samples. It incorporates a high-speed blending system which homogenizes the sample first, then an automatic Karl Fischer titration takes place. The blending can be timed from 0 to 15 min, coping with almost any kind of sample which normally has to be manually ground.

*For further information contact Baird & Tatlock, PO Box 1, Romford RM1 1HA, UK. Tel.: 01 590 7700.*

Circle No. 12 on Reader Enquiry Card

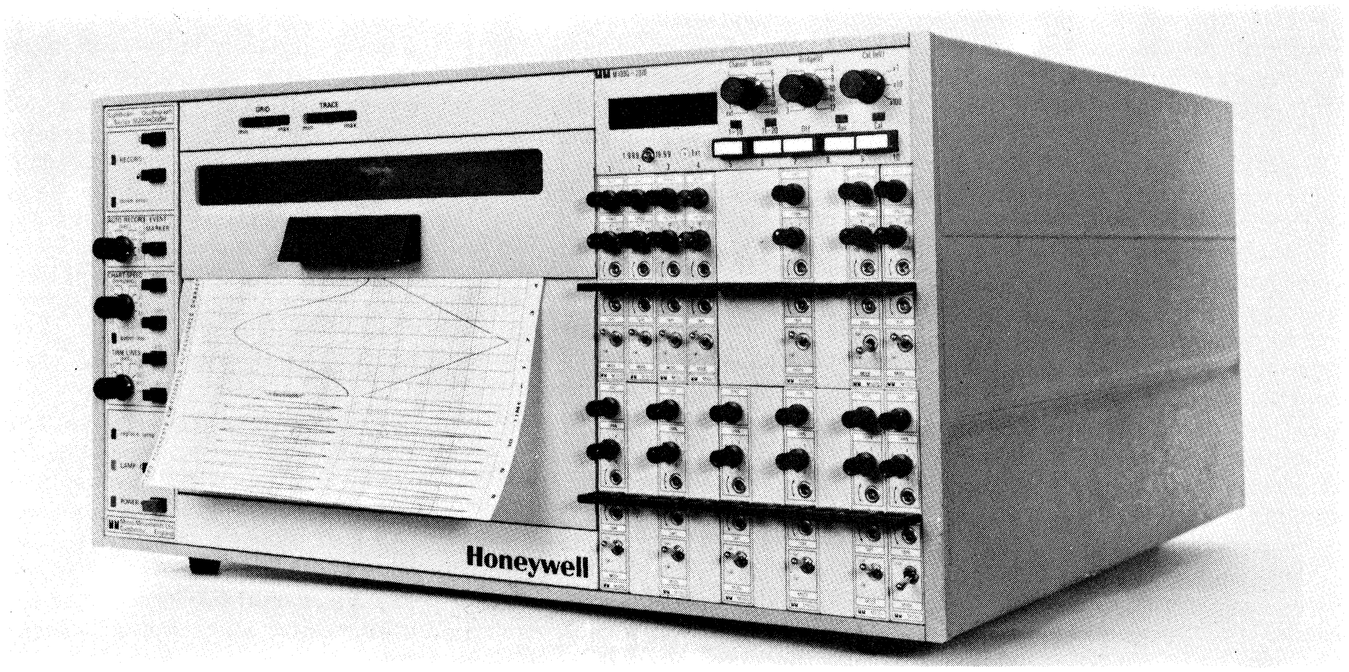

*Honeywell Control Systems have launched a new family of British-made and designed oscillograph recorders. The smallest unit is a portable 10-channel instrument for field or laboratory use; and at the top of the range is a 30 cm chart width instrument with up to 30 channels and a very wide chart speed range, which should meet the exacting requirements of laboratory engineers and technicians in providing high-quality hard-copy records. A unique feature of the range is the wide selection of on-board signal conditioning modules which give unprecedented flexibility for matching directly to almost any type of signal source. Included are modules to interface directly with virtually any transducer, active filters, transient capture and alphanumeric printing with keypad or remote-entry facilities. (Contact Honeywell Control Systems Ltd at Charles Square, Bracknell, Berkshire RG12 1FB, UK, for more information.)*

Circle No. 13 on Reader Enquiry Card

### Mettler DL 40

Several applications of the Mettler DL 40 Memotitrator have been described to *JAC* by MSE Scientific Instruments. Firstly, the company suggests using the DL 40 in conjunction with a platinum redox electrode to determine chemical oxygen demand (COD)—results are presented calculated in COD units. Secondly, they draw attention to a paper in *Analytical Chemistry* which describes the Memotitrator and an Orion fluoroborate electrode to automate determinations of anionic and cationic detergents. Finally, the titrator is proposed as a method for analysing the purity of silver.

*Full details from MSE Scientific Instruments, Manor Royal, Crawley, West Sussex RH10 2QQ, UK. Tel.: 0293 31100.*

Circle No. 14 on Reader Enquiry Card

### Moisture determination

A variety of products (food powders, fruits, cereals, pharmaceuticals, adhesives etc.) have to be tested during processing, packaging or storage for moisture contact. Dynatech are marketing an accurate alternative to conventional drying methods which can cut the time taken by 75% or more.

The Artek 'Accudry' uses a digitally controlled infra-red radiation drying oven and cooling chamber with a system temperature range from 5°C above room temperature to 150°C; the Accudry's method of heating enables products to be dried completely at temperatures which are often lower than those needed with conventional ovens. And up to 12 samples can be dried, with accuracies of up to 0·1%, or better, when compared with conventional drying ovens.

*Full information from Dynatech Laboratories Ltd, Daux Road, Billingshurst, Sussex RH14 9SJ, UK. Tel.: 040381 3381.*

Circle No. 15 on Reader Enquiry Card

### Laboratory Impex

There is now an 'intelligent' printer available for Laboratory Impex's CCVI modular haematology system. The printer can calculate MCH and MCHC; it 'converses' with the CCVI through a 16-position keyboard. All calculated and measured parameters are printed-out on standard 40-column printer paper. Results from up to 100 patients can be memorized and then displayed in one of three formats: print-out during operation, print-out in a condensed form as a daily report or a complete haematology profile of each patient with date, identification number and ward. The CCVI printer is simple to operate.

*Further details are available from Laboratory Impex Ltd, Lion Road Twickenham, Middlesex TW1 4JF, UK. Tel.: 01 891 4881.*

### *Cell sorting*

Laboratory Impex is marketing the Becton Dickinson cell sorters and analysers in the UK. Current applications of the FACS series include routine immunofluorescence, cell cycle analysis and cell type differentiation.

Circle No. 16 on Reader Enquiry Card

### PV 8350 extras

A series of new features has been announced for Philips's modular PV 8350 vacuum spectrometer system. For example the spectrometer optics can now include certain analytical lines at longer wavelengths than the normal limit of the spectrometer: with the standard 2160 line/mm grating, besides covering the range 177 to 410 nm at a dispersion of 0·46 nm/mm in the first order, the lines Na

588·9 nm and Li 610·3 nm can be measured in the first order. Exit slits for these lines are mounted on the Rowland circle on the opposite side of the entrance slit to the normal exit slit block. Compromises associated with expanding the wavelength range by measuring lines in higher orders of the grating are thus avoided.

For metal analysis, a new 50 Hz source (PV 8560) combines the advantages of monoalternance excitation with computer-controlled high-energy pre-burn for installations where the high speed of the Philips PV 8530 500 Hz source is not required. By localizing pre-melting, the pre-burn minimizes problems caused by sample inhomogeneity.

For systems which include an inductively coupled plasma source unit, a large number of accessories are now available—for instance automatic background correction and a choice of automatic systems for handling batches of solutions, dilution of samples or measurement by multi-element standard additions.

Advanced interface electronics extend the dynamic range of measurement to 300 000 : 1; this is particularly valuable in ICP analyses and also allows full advantage to be taken of the measurement capabilities of lines used for spark analyses.

Two comprehensive software packages are offered: Cespec 7 for the Philips P 851 computer with fixed and flexible disc storage and Cespec 8 for the HP 9825 desk-top computer. Software functions include spectrometer control, result calculation, calibration and inter-element correction by regression and tolerance checks of analyses and material compositions.

*Details from Pye Unicam Ltd, York Street, Cambridge CB1 2PX, UK. Tel.: 0223 358866.*

**Circle No. 17 on Reader Enquiry Card**

### Flexibility, reliability, economy—SRA-2000

The SRA-2000 system is now ready for delivery. It is built around two analytical subsystems: a Technicon SMAC II and a Technicon RA-1000, linked by a common computer command module. Technicon claim the following advantages for their new product:

efficient sample and data handling;
efficient and cost-effective reagent usage;
efficient sample utilization;
discretionary analysis and reporting;
in-built analytical support;
simple handling of paediatrics;
in-built quality-control procedure;
composite result reporting;
flexible data storage and retrieval.

*Details from Technicon Instruments Company Ltd, Evans House, Hamilton Close, Basingstoke, Hampshire RG21 2YE, UK. Tel.: 0256 29181.*

**Circle No. 18 on Reader Enquiry Card**

### PRTs

The 6800 and 6900 platinum resistance thermometers incorporate Comark Electronics' proven microprocessor-controlled A-D system, auto-zeroing, auto-calibrating and exceptional scale length. They are described as accurate, stable, versatile, easy to operate and competitively priced.

The 6800 will accept DIN plug inputs from up to five A or B type PRT probes, any one of which can be selected by a rotary switch on the front panel to give a read-out on the 14 mm LED display. Push-button selected scales allow the display to be shown in degrees absolute, Celsius or Fahrenheit and directly in Ohms. The range of the Model 6800 is from $-200°$ Celsius up to $+700°$ Celsius at an overall accuracy of 0·1° Celsius and resolution down to 0·01 of a degree. An array of push-buttons on the front panel controls all functions and modes and allows the entry of high/low alarm limits and refinements such as a factor to scale and offset the readings. The alarms also provide an output for control purposes. Parallel BCD or two-range analogue outputs are provided.

The 6900 accepts inputs from either one A or B type PRT probe or one thermocouple. Of the latter, types K, J, T, R or S can be accommodated, being selected by using the front-panel push-buttons; these controls also allow the entry of alarms and factors as in the 6800. The LED will display in degrees absolute, Celsius or Fahrenheit, microvolts or Ohms. Depending upon the type of thermocouple in use, the range of the 6900 is from $-200°$ Celsius up to $+1760°$ Celsius and, on the microvolts scale, from $-10$ to $+75$ millivolts. Resolution is to 0·1 of a degree in the thermocouple mode and 0·1/0·01 of a degree for the PRT mode. A three-range analogue output is provided.

The PRT section of these instruments is characterized to DIN 43760 (1980); thermocouples should be constructed of materials to BS 4937.

The instruments are housed in compact and robust cabinets which incorporate integral carrying handles that double as tilt stands. They measure 254 × 325 mm, the longest dimension being with the handle in the extended position. Supply voltage requirements are either 110 V or 220 V.

*Comprehensive leaflets including full specifications are available from Comark Electronics Ltd, Rustington, Littlehampton, West Sussex BN16 3QZ, UK. Tel.: 09062 71911.*

**Circle No. 19 on Reader Enquiry Card**

### Interfaces/sampler

The Varex Corporation of Rockville, USA recently launched two interfaces and an automatic sampling system. One interface, the 'Simple', is designed for fast data acquisition with microcomputers; the other, the 'Universal', can acquire, digitize and store data from most analytical instruments and can be easily connected to the majority of computers.

Varex's Automatic Sampling System is under microprocessor control and gives virtually contamination-free injections of samples. Up to 90 samples can be injected in any selected sequence, at any volume, for any number of times. It can be programmed to mix different volumes of different samples for the same injection, and rinse the sampling valve between injections. Samples are injected directly into the injection valve, reducing sample loss and band broadening to a minimum. The syringe is automatically washed before drawing a sample. Ten injection programs, with up to 100 sequences can be stored in permanent memory for future use.

*More information from Varex Corporation, 12221 Parklawn Drive, Rockville, Maryland 20852, USA. Tel.: 301 984 7760.*

**Circle No. 20 on Reader Enquiry Card**

### Emission spectrometer

The ICP/6000 system is a high performance, inductively coupled plasma emission spectrometer. It is described by Perkin-Elmer as offering high speed, precision, flexibility and operating simplicity for multi-element trace metal analysis. Controlled by a new laboratory computer and dedicated ICP software, the system is capable of analysing samples sequentially at a rate of more than 20 elements/min.

The spectrometer was designed to handle the widest possible range of sample matrices. Each element is analysed under optimum spectral conditions: a dual-grating monochromator ensures high resolution over the entire wavelength range (175–900 nm); each grating is used only in the first order for high energy throughput; and computer control of wavelength selection ensures accurate and stable calibration. Furthermore, the optical system can be purged with an inert gas, so analyses in the far UV region can be performed.

The complete sample-introduction assembly is extremely resistant to corrosion so that the system can be used to aspirate any laboratory acid, as well as high levels of dissolved solids. The cross-flow nebulizer and spray chamber are designed to give high analytical precision without adjustment. The plasma torch is demountable for low running costs and easy maintenance, with a choice of quartz or alumina sample aerosol tubes.

The software is resident on the hard disc of the computer. At the start of an analysis, the operator simply types 'ICP' and is then guided by prompts. Soft keys simplify parameter entry for method development and up to 108 individual element files can be stored in a single method file.

High-resolution colour graphics allow the analyst to easily evaluate and compare spectra, since blanks, standards and samples are displayed and printed in their own designated colours. Each of the seven modes of operation is colour-coded.

The data generated during an analysis are stored automatically on hard disc and can be reported in a chosen format as hard copy or transmitted to a floppy disc or data-bank for archiving. When the ICP/6000 is not in use the computer can be used as a stand-alone unit.

*Contact Perkin-Elmer Ltd, Post Office Lane, Beaconsfield, Buckinghamshire HP9 1QA, UK; tel.: 04946 6161 for more information.*

**Circle No. 21 on Reader Enquiry Card**

### Gas chromatographs/mass spectrometers

The NERMAG gas chromatographs, mass spectrometers and accessories are discussed in a brochure which is available from the UK distributor: Dorand Electronics.

*Requests to Dorand Electronics Ltd, Allens Lane, Hamworthy, Poole, Dorset BH16 5DA, UK. Tel.: 0202 622006. Enquiries outside the UK to SNG, NERMAG, 49 Quai du Halage, 92500 Reuil, Paris, France.*

**Circle No. 22 on Reader Enquiry Card**

### Electronic counter

Rather that simply counting input pulses, a Trumeter counter can be programmed on site to convert pulses into units of measurement. Four external thumbwheel switches on the recently launched unit allow a four-figure multiplication factor to be programmed in; the counter display is then the product of the input pulses and the multiplication factor.

The multiplication factor is determined simply by a trial run; for example comparing the previously measured length of a piece of continuous sheet material to the number of pulses produced by a transducer attached to the production machine roller. Where continuously produced materials are then cut into units, a multiplication factor could be programmed to enable the counter to display the number of separate sheets. Other applications include compensation for known errors or deviations, metric/Imperial conversions etc.

This new Trumeter counter is their Progammmable Add and Subtract Counter, which will count up or down and is coded LCD4 Xn. It has a four-digit 12 mm high LCD display, a count speed of up to 500/s and a DIN-sized panel mounting case. Rear screw connections are provided for 12 V d.c. supply, input devices and external resetting. Either electronic or contact-closure input devices may be used. The counter has an internal rechargeable battery back-up of 1000 h.

*Technical literature is available from Trumeter Company Ltd, Radcliffe, Manchester M26 9NX, UK. Tel.: 061 724 6311.*

**Circle No. 23 on Reader Enquiry Card**

### U-V

Ultra-violet units with a large exposure area have tended to be expensive; Decon Laboratories have launched a unit with an exposure area of 45 x 24 cm, which is priced at £97·00. The light source is four 15 W actinic blue fluorescent tubes backed up by a highly effective reflector and controlled by a 6 min electromechanical timer. This is housed in a cabinet which has a foam pad attached to its spring-loaded lid to ensure even distribution of pressure over the glass plate.

To complement this unit Decon Laboratories are also offering a range of spray-coated photo-sensitive copper boards. These are available in four standard sizes, as single sided or double sided with fibre-glass or pertinax bases. The developer for these boards is granular and is packed in small sachets which make up a 0·5 l solution.

*Details from Decon Laboratories Ltd, Conway Street, Hove, Sussex BN3 3LY, UK. Tel.: 0273 739241.*

**Circle No. 24 on Reader Enquiry Card**

### Change of address

Busch (UK) Ltd, suppliers of vacuum pumps and compressors, have moved to Vulcan Way, New Addington, Croydon, Surrey CR0 9UG, UK.

**Circle No. 25 on Reader Enquiry Card**

### Analytical Data Manager

*Letter from Alan Gow (President and CEO: Interface Design Inc.) to Peter Stockwell (Ed.)*

Enclosed is some information on our new computer-based spectrometer control system. It was introduced at the Pittsburgh Conference; hopefully you can use it in your Product News Section.

Ironically, our Analytical Data Manager—ADaM—addresses a number of the issues you raised in your editorial in *Journal of Automatic Chemistry's* January–March 1983 issue. Until our ADaM was available, all prior attempts to automate the Beckman/SpectraMetrics (Beckman/SpectraMetrics, Inc., 204 Andover Street, Andover, Massachusetts 01810, USA) spectraspan have been with passive microcomputers. First with the Tektronix 4052 and then with their own DataSpan—both in the passive mode (just handling the data).

The ADaM takes a different approach, one we believe will become the dominant method. We have developed an interactive interface between the spectrometer and microcomputer. The interface includes its own microprocessor, RAM and I/O capabilities, and controls the acquisition of raw data as well as the microcomputer and peripherals.

Thus, the specific microcomputer that is used becomes less important. In fact the system can be adapted to use the latest microcomputer available and avoid becoming obsolete. The initial ADaM included the Commodore 8096. We now offer the IBM-PC as an option. We are

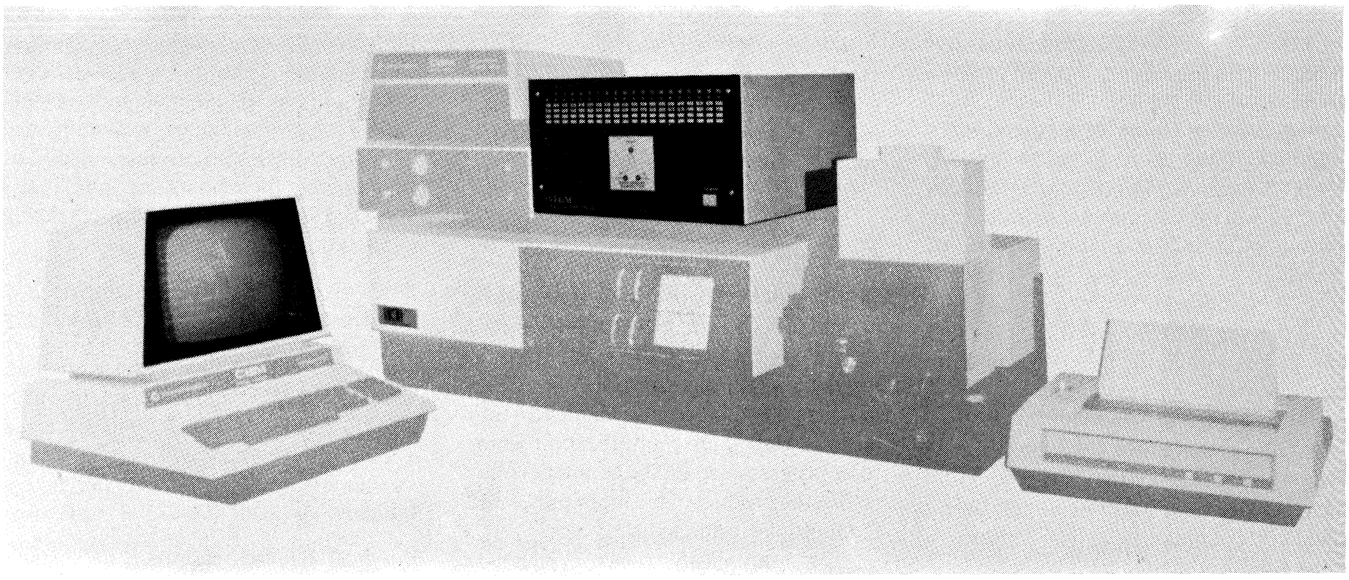

*ADaM. Productivity can be increased at least five times by, for example, fast and accurate peaking on the exact wavelength using CRT display; simultaneous 20-channel dynamic background correction; 20 profiled channels instantly available for any sample; fewer dilutions required because the electronic dynamic range is increased 200X; easy retrieval of profiles and calculated data. Results are better because ADaM's advanced electronics make full use of the SpectraSpan optics. Operation is user oriented.* (*Interface Design Inc., 6116 Skyline Drive, Suite 106, Houston, Texas 77057, USA; tel.: 713 783 3607.*)

**Circle No. 26 on Reader Enquiry Card**

evaluating others and may offer another option before the end of this year.

Another important feature is the access to the software we give the customer. Regardless of how thorough we are, we cannot possibly design the software to meet all laboratory needs. With the ADaM, the chemist can customize the programming (to the extent of his programming ability or resources) to fit his own needs.

Of course, not all analytical instruments are as complex as a spectrometer, but the concept is the same: a versatile, interactive, intelligent interface. More expensive initially perhaps, but much more cost effective in both the short and long term. Our increased productivity claim of five to 10 times is not a casual advertisting statement.

We now offer other options including a graphics plotter (HP7470A), (Hewlett-Packard, Dept. 299A, Corvallis, Oregon 97330, USA), an X-Y-Z autosample changer, and a FiAtron (FiAtron Systems, Inc., 6651 N. Sidney Place, Milwaukee, Wisconsin 53209, USA) SHS-300 flow-injection system.

The points made by several people in your Comments Section (of the same January–March 1983 issue), with regard to just beginning to learn the potential of FIA, are quite true in our estimation. The combination of the echelle grating, multichannel, plasma spectrometer with the ADaM and a full FIA system should open the opportunity to develop applications at a fast rate.

### Hydrogen safety

The use of hydrogen as a carrier gas in chromatography has the advantage of fast analysis times—it is twice as fast as helium and four times faster than nitrogen. The optimum linear velocity (giving the highest plate count) is much bigger for hydrogen than for the other gases. There is only one serious disadvantage: the danger of explosion if hydrogen leaks into the ambient air. Even with a small leak the explosive limit of 4% can easily be reached in the oven compartment of a gas chromatograph.

Chrompack developed the Hydrogen Safety System to monitor the actual hydrogen concentration in gas chromatographs and to take the required precautions if the hydrogen concentration becomes a fraction of the explosive limit. The concentration at which gas flow and oven heating are switched off can be set by the operator. The main parts in the safety system (the detector, the sampling pump and the electromagnetic valve) are continuously checked for faults by an electronic circuit.

*Further information from Chrompack Nederland B.V., PO Box 3, 4300 AA Middelburg, The Netherlands. Tel.: 01180 11251.*

**Circle No. 27 on Reader Enquiry Card**

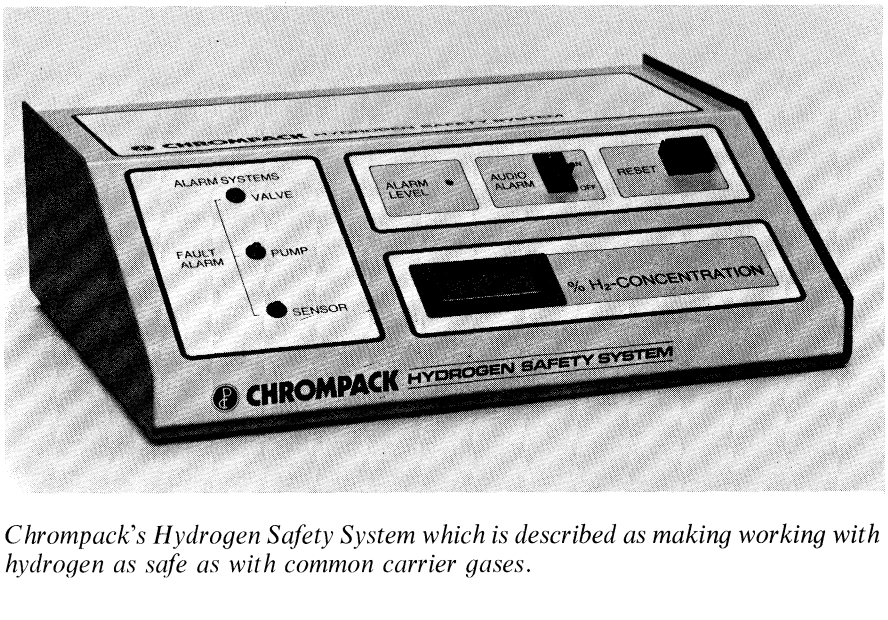

*Chrompack's Hydrogen Safety System which is described as making working with hydrogen as safe as with common carrier gases.*

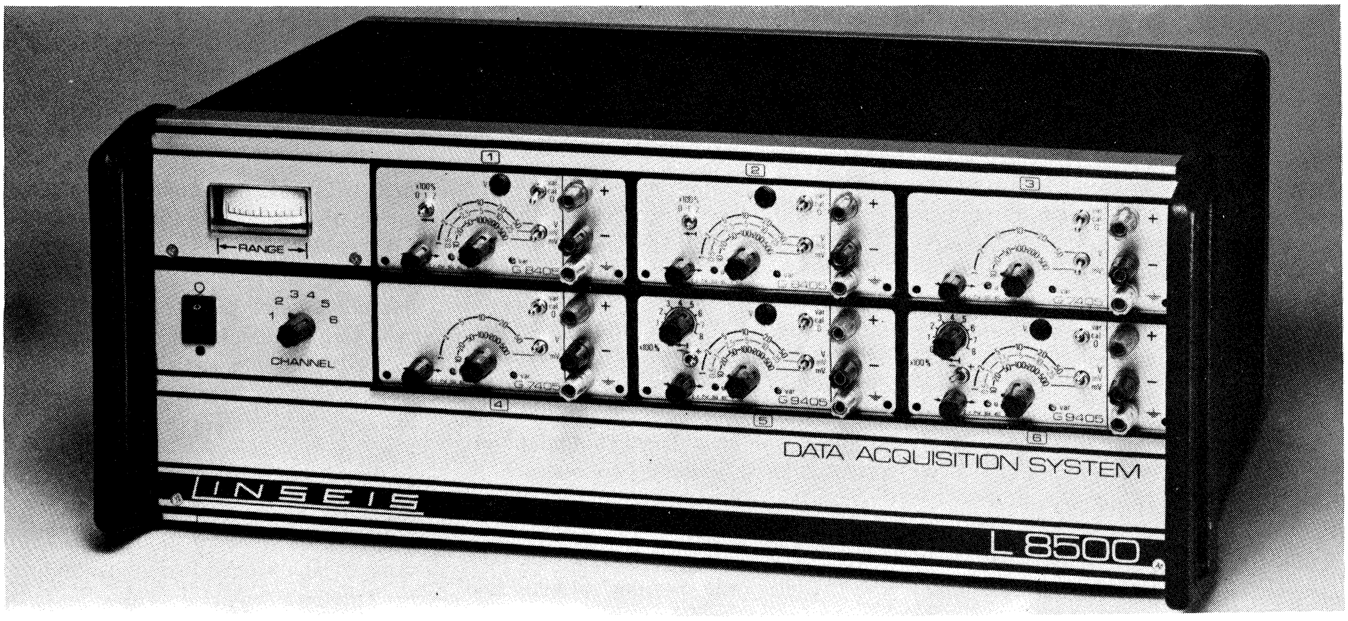

*Linseis's L8500 Data Acquisition System. It will accept one to six signals and consists of a basic housing plus plug-in modules (the measuring input modules from the company's upright recorder series L2005). Signals are taken up by the modules, standardized, digitized and passed to the computer via an interface (V24) or an IEC bus. Before the L8500 one digital instrument had to be used for each signal. To adapt the instrument to different signals it is not necessary to buy a new digital instrument, rather all that is needed is a new input module. Full explanation from Linseis GmbH, Werk Selb, Vielitzer Strasse 43, D 8672 Selb, FR Germany.*

**Circle No. 28 on Reader Enquiry Card**

### Thermal data

A cost-efficient approach to thermal analysis, Mettler's FP800 Thermosystem consists of a central control unit and five different measuring cells. The system will supply all the data needed for thermal analysis: melting, boiling and clouding temperatures, and dropping and softening points. The system also enables the heat of transformation to be determined, and samples can be investigated under thermal microscopy conditions. The measuring cells of the FP800 offer the user several system-design options; it is not necessary to buy all the modules at once so a thermal analysis system can be put together as needs dictate. Peripheral instruments such as a printer, a recorder and a computer can all be connected—the necessary data interfaces are built into the control unit.

To operate the FP800, the user simply proceeds by following a dialogue with the control unit: this can be done in English, French, German, Italian or Spanish.

*Details from Mettler Instrumente AG, CH 8606 Greifensee, Switzerland.*

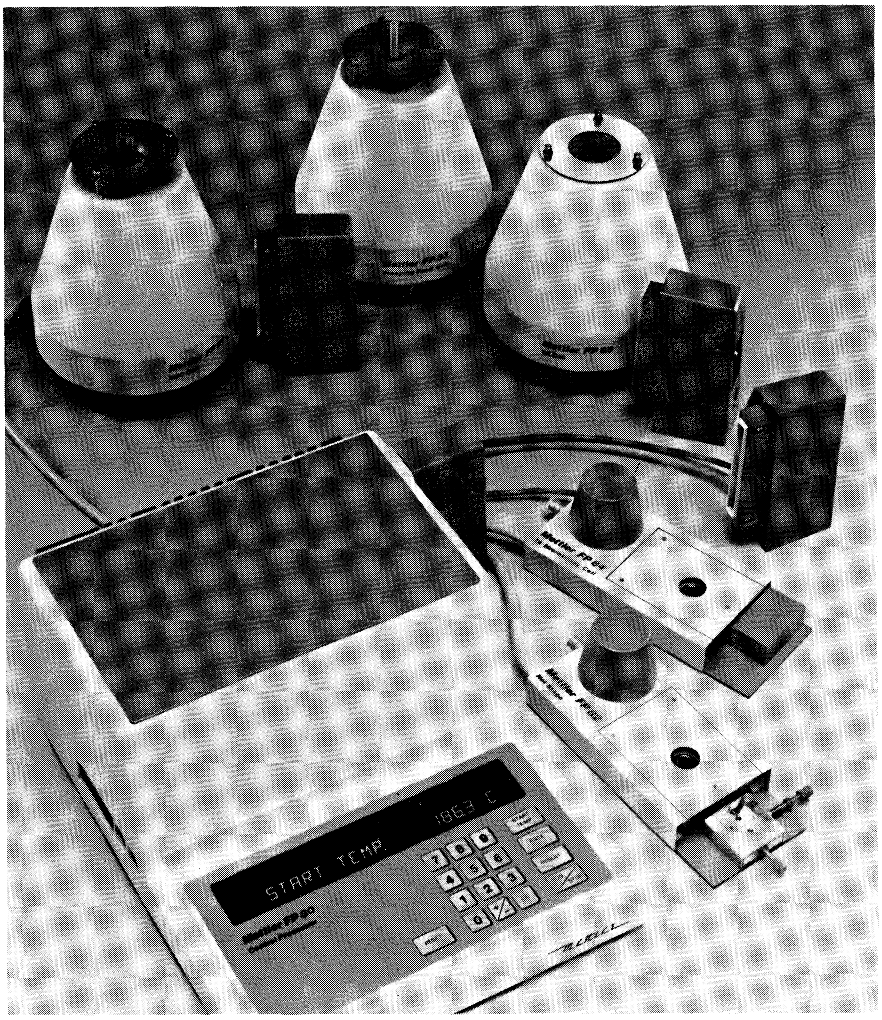

*Thermal analysis has now become essential in production and quality control, also in research and development—Mettler's FP800 provides cost-effective, user-oriented information. (Mettler Instrumente AG, Greifensee, Switzerland.)*

**Circle No. 29 on Reader Enquiry Card**

### Platelet aggregation

A computerized platelet aggregation system is available from Payton through their sole UK distributor, Centronic Sales Ltd. Using an Apple II microcomputer, Payton have produced a system which can be linked not only to their own range of aggregation modules, but also to any other make or model of instrument. The software package allows inducer concentrations to be automatically computed for precise final concentrations, and provides audible and visual prompting for all user tasks. A variety of data manipulations can be performed, and corrections and alterations entered as appropriate. For later retrieval, reproduction or analysis, all data is stored on a diskette. Patients' results are provided in hard copy by means of any suitable 80-column graphics printer. The data, which is presented in an easily readable form, can then be attached to medical records.

The system provides fast results for a number of standard assays, such as Von Willebrand, and, if required, Payton will develop an exact software package to meet specific aggregation needs.

*More information from Centronic Sales Ltd, Centronic House, King Henry's Drive, New Addington, Croydon CR9 0BG, UK. Tel.: 0689 47021.*

Circle No. 30 on Reader Enquiry Card

### Turbidity measurement

The Model 40-100 measures turbity in drinking-water. The nephelometer is EPA approved and provides a digital read-out directly in nephelometric turbidity units (NTUs). The wide range, 0–9·99 and 0–99·9 NTUs, is appropriate for both raw and finished water studies. The 40-100 has a sample viewing port which prevents lint, dirt or fingerprints from creating high readings. Capped sample bottles eliminate bubbles or contaminants in the sample. Operation is easy and dependable with low drift; it is readable to 0·01 NTUs on the 0–99·99 NTU range and to 0·1 on the 0–99·9 range, stray light is under 0·03 NTUs. No volume measurement is required and a sample is approximately 20 ml; it has standard recorder outputs.

Accessories are available, including a continuous-flow attachment. The nephelometer comes complete with operating and service manual, three sample cells, two spare fuses and one spare lamp.

*More information from Tekmar Company, PO Box 371856, Cincinnati, Ohio 45222, USA.*

Circle No. 31 on Reader Enquiry Card

### Industrial pH/Redox meter

A range of low-cost pH/Redox meters has been announced by Kent Industrial Measurements Ltd. The meters are designed for continuous measurement of pH levels in industrial liquids. With a digital display, and a choice of several control, alarm and output options, the panel-mounting Kent EIL 9140 Series instruments are suitable for on-line applications in a number of industries. They can be connected to chart recorders, controllers, data loggers or computerized control systems, and can be used to provide two- or three-step automatic pH control using one or two reagents to adjust the acidity or alkalinity of the test liquid.

A variety of dip-and-flow type electrodes, electrode cleaning systems and control valves is available, enabling complete pH/Redox measurement and control schemes to be implemented.

There are four models. The basic model, the 9141, gives pH indication only, and is equipped with non-isolated current output. The 9142 model has the same features plus two on-off control/alarm points. The next model, the 9143, has in addition an isolated current output. On the most sophisticated model in the range, the 9144, one of the control/alarm points is fitted with a mark-space control function to facilitate the monitoring or continuous dosing systems with long time constants. All instruments are housed in a DIN-standard case measuring 96 × 96 × 160 mm, and share the same 0–14 pH or 0 to ±1999 mV range, 0·01 pH discrimination, and stability of better than 0·02 pH in 24 h at constant temperature. They all incorporate temperature compensation over a range of 0–100°C. This can be automatic, with a Pt100 temperature compensator installed in the electrode system, or fixed at 10° intervals by placing a fixed resistor of the appropriate value in the temperature-compensation circuit. Various current output ranges are available, giving 0–10 mA, 0–20 mA or 4–20 mA, in isolated or non-isolated versions. The output current can be expanded to cover a pH span down to 5 pH, pre-set anywhere in the range 0–14 pH. High and low alarms, with either normal or failsafe operation, are set by means of screwdriver-operated controls on the front panel of the instrument.

*More information from Kent Industrial Measurements Ltd, Hanworth Lane, Chertsey, Kent KT16 9LF, UK. Tel.: 09328 62671.*

Circle No. 32 on Reader Enquiry Card

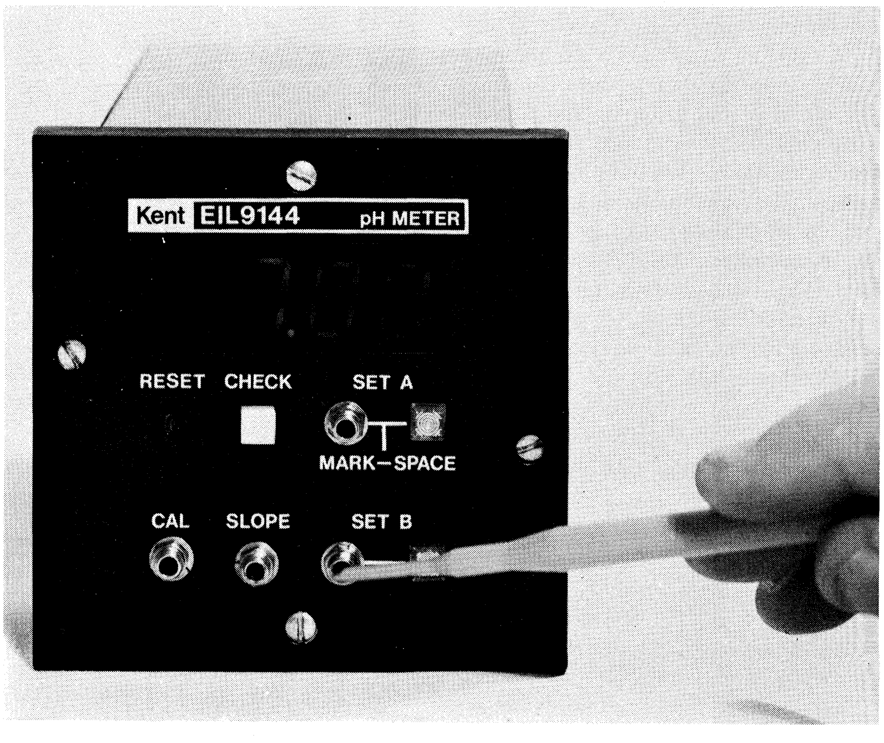

*Series 9140 pH/Redox meters are described as having 'potential applications whenever there is a need for a panel-mounting instrument to provide continuous analysis/control of industrial liquids'. The most likely applications include brewing, chemical manufacture, dairying, effluent treatment, food and drink processing, metal finishing and plating, paper and board manufacture. (Kent Industrial Measurements, UK.)*

